# Bioinformatics study of the pharmacological mechanism of sodium-glucose co-transporter 2 inhibitors in type 2 diabetes mellitus and coronary heart disease based on network pharmacology

**DOI:** 10.1097/MD.0000000000047306

**Published:** 2026-01-30

**Authors:** Shuai Yu, Jingru Li, Xinyu Wu, Si Lu, Chaguo Li, Huan Cheng, Hao Guo, Ping Yang, Luqiao Wang

**Affiliations:** a Department of Cardiology, The First Affiliated Hospital of Kunming Medical University, Kunming, Yunnan, Peoples R China; b Zhumadian Central Hospital, Zhumadian, Henan, China.

**Keywords:** coronary heart disease, network pharmacology, sodium-glucose co-transporter 2 inhibitors, type 2 diabetes mellitus

## Abstract

Sodium-glucose co-transporter 2 inhibitors (SGLT2is) have consistently been shown to be beneficial in reducing the incidence rate of coronary heart disease (CHD) in diabetic patients. However, their specific pharmacological mechanisms are still unknown. Therefore, we intend to explore the mechanism of SGLT2is in patients with type 2 diabetes mellitus (T2DM) combined with CHD through a network pharmacological approach. Firstly, the Swiss Target Prediction and Drugbank databases were used to predict the targets of SGLT2is. The CHD dataset GSE113079 and T2DM dataset GSE118139 were downloaded from the gene expression omnibus database, and the differentially expressed genes (DEGs) were analyzed. Then, the predicted targets of SGLT2is intersected with the DEGs of the two diseases, and the results were used to construct the “drug-targeted-disease (D-T-D)” and protein–protein interaction networks. Gene Ontology functional analyses and Kyoto Encyclopedia of Genes and Genomes pathway were used for functional studies of target genes. Finally, molecular docking of SGLT2i with target proteins was performed using AutoDockTools software, and the docking results were verified by ELISA, RT-qPCR and western-blot experiments. Among the drug targets of SGLT2is and DEGs in T2DM and CHD, a total of 14 common gene targets exist, and these genes mainly affect the disease progression through the “chemical carcinogenesis-reactive oxygen species”,“apoptosis,” and “chemical carcinogenesis-receptor activation” pathways. Through hub gene identification, we identified 11 hub genes from these 14 targets, of which epidermal growth factor receptor (EGFR) had the highest score. Molecular docking results showed that ertugliflozin had the strongest intermolecular binding to EGFR and that ertugliflozin improved the viability of high-glucose (HG)/high-lipid combined hypoxia-reoxygenation-injured (HG/HP + H/R) cardiomyocytes and inhibited EGFR expression in cardiomyocytes. Based on network pharmacology and bioinformatics analysis, our study elucidated that EGFR is the hub gene for T2DM combined with CHD myocardial injury. Molecular docking and cell experiments further confirmed that SGLT2i-ERTU improves HG/HP + H/R myocardial cell injury by targeting EGFR. Our study deepened the pharmacological mechanism of SGLT2is in the treatment of T2DM combined with CHD and provided a new perspective and therapeutic basis for future experimental research and healthcare.

## 1. Introduction

Coronary heart disease (CHD) is a myocardial ischemic disease caused by lipid plaque formation and luminal narrowing in the coronary arteries, which severely impairs the coronary blood flow and cardiac function of patients. Type 2 diabetes mellitus (T2DM) is a metabolic disease characterized by chronic insulin resistance. Due to the body’s insulin resistance and insufficient insulin release, the body’s sugar and lipid metabolism is abnormal,^[1]^ which eventually complicates a series of organ damage, such as the heart and kidneys.^[2]^ Existing studies have shown that T2DM patients may experience cardiovascular events up to 4 times more frequently than healthy people; moreover, compared to nondiabetics, diabetic patients with myocardial infarction (MI) have a greater death risk.^[3,4]^ All these illustrate the damage of diabetes to the organism, especially the cardiovascular system.

Sodium-glucose co-transporter 2 inhibitors (SGLT2is) are a novel oral hypoglycemic agent that significantly reduces the risk of death in patients with diabetic.^[5]^ In the organism, SGLT2is impair the glucose uptake capacity of renal tubular epithelial cells by decreasing the activity of SGLT2 in the proximal renal tubule, which in turn promotes urinary glucose excretion and achieves the reduction of plasma glucose concentration.^[6]^ However, recent studies have shown that SGLT2is not only have an important role in diabetes treatment but also reduce the incidence of adverse cardiovascular events and delay the progression of CHD.^[7]^ Terasaki et al have demonstrated that SGLT2i–dapagliflozin–can prevent atherosclerosis by lowering glucose and thereby inhibiting the formation of monocyte-macrophage foam cells.^[8]^ Not only that, SGLT2is also have an effect on the reduction of the incidence of adverse cardiovascular events.^[5]^ Currently, the U.S. Food and Drug Administration (FDA) has approved 4 different types of SGLT2is (including dapagliflozin, empagliflozin, canagliflozin, and erpagliflozin) for the treatment of T2DM.^[9]^ However, the specific mechanism of cardiovascular protection by SGLT2is in patients with T2DM combined with CHD remains unclear. Therefore, in this article, we will explore the specific mechanism by which SGLT2is exerting cardioprotective effects in patients with T2DM and CHD.

Network pharmacology was first proposed by Hopkins in 2007. It aims to effectively explore drug targets and pathway relationships from a biological network perspective by combining Multi-omics data analysis, computer technology and online databases using bioinformatics analysis techniques.^[10–12]^ Compared with traditional experimental pharmacological methods, network pharmacology can more effectively explore the relationship between diseases and drugs from the perspective of biological networks. On the data level, network pharmacology integrates compound targets, disease-related genes, pathways and other databases; on the analysis method, network pharmacology integrates network analysis and topological features to predict key targets, and groups drugs, targets, diseases and other elements of biological networks into a whole, and through the synergistic effect of “multi-targets, multi-pathways,” avoiding the limitations of single targets in traditional research.^[13,14]^

Complementing network pharmacology, molecular docking can further validate the binding between the drug and the target gene. Molecular docking is a computational simulation technique used to predict the optimal binding conformation and intensity between small-molecule compounds (*e.g.*, drug components) and biological macromolecules (*e.g.*, proteins).^[15]^ The core principle is to model intermolecular interactions (*e.g.*, hydrogen bonding, hydrophobic interactions, electrostatic interactions, etc) and evaluate their binding potential.^[16]^ Currently, the method of analyzing the potential targets of the main components of drugs in the treatment of diseases through network pharmacology is commonly used in diseases. For example, Li et al explored the potential mechanism of Huanglian-Jiedu-decoction in preventing sepsis based on the strategy of network pharmacological prediction and molecular docking;^[17]^ Han et al explored the mechanism of Panax notoginseng in the treatment of myocardial fibrosis using network pharmacology.^[18]^ However, there are fewer studies on the network pharmacology of SGLT2is in diabetes combined with CHD. Therefore, we use network pharmacology to predict the target of SGLT2is in diabetes with coronary heart disease, and understand the biological basis of drugs and diseases, analyze the biological pathways involved, which will lay a foundation for us to explore their ingenuity further.

**Figure 1.**
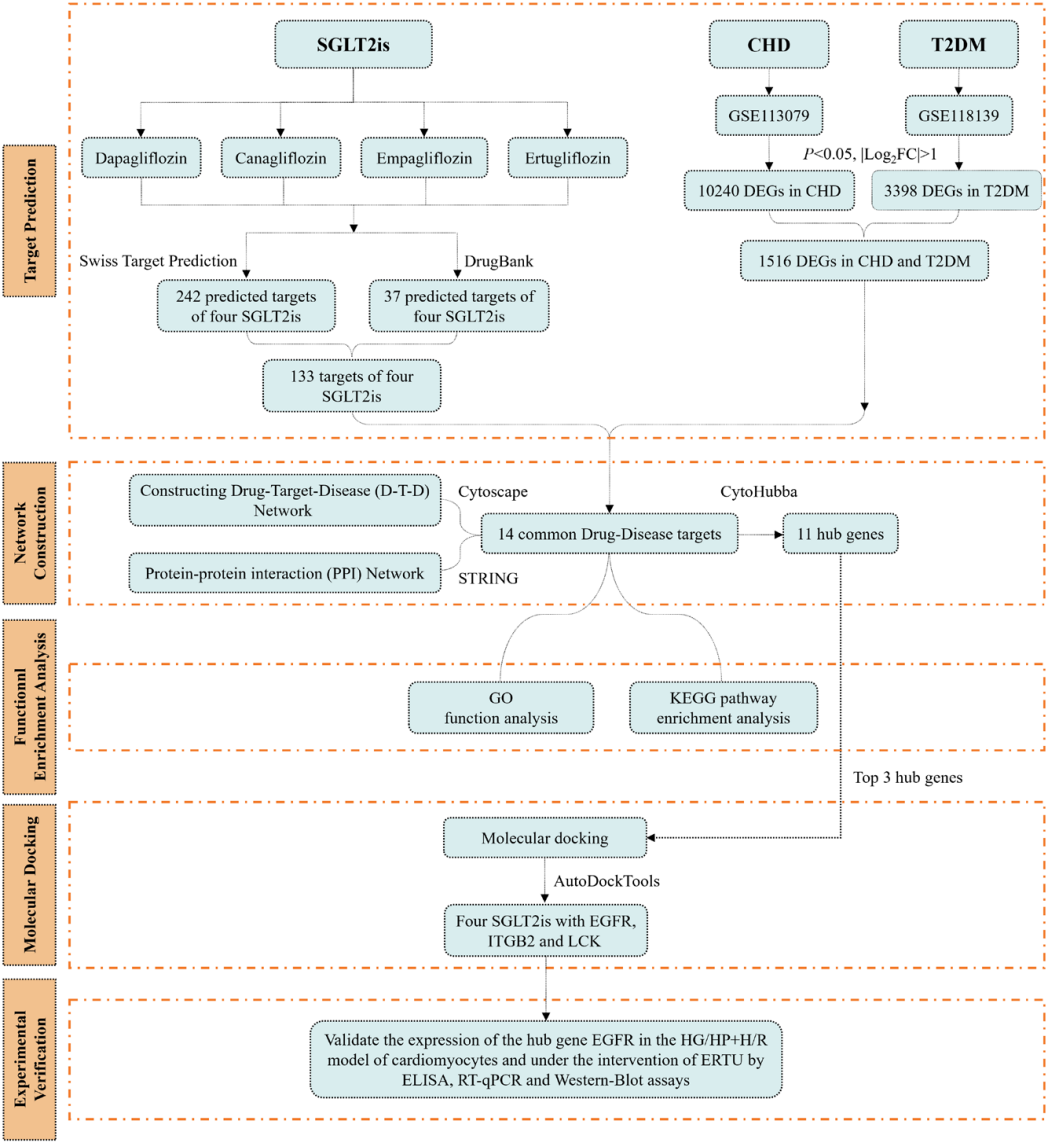
The overall flow of this study. SGLT2is: codium-glucose co-transporter 2 inhibitors. CHD = coronary heart disease, DEGs = differentially expressed genes, ELISA = enzyme-linked immunosorbent assay, ERTU = ertugliflozin, HG/HP + H/R = high-glucose/high-lipid combined hypoxia-reoxygenation, ITGB2 = Integrin subunit beta-2, LCK = LCK proto-oncogene, Src family tyrosine kinase, RT-qPCR = real-time quantitative PCR, T2DM = type 2 diabetes mellitus.

In this study, we combined the network pharmacology approach with Gene Expression Omnibus database for drug-target prediction and gene network analysis. Firstly, we screened the relevant targets of SGLT2is from PubChem and Drugbank databases. From the GEO database, we obtained the diabetes dataset GSE118139 and the coronary heart disease dataset GSE113079, and identified differentially expressed genes (DEGs) associated with the two diseases. Subsequently, the predicted targets of SGLT2is were intersected with the DEGs, and the results were visualised as a “drug-target-disease (D-T-D)” network using Cytosccape. Metascape was used for the Kyoto Encyclopedia of Genes and Genomes (KEGG) pathway and Gene Ontology (GO) biofunctional analysis of the targets. Finally, we confirmed the potential role of SGLT2is in improving myocardial injury in CHD combined with T2DM through molecular docking and in vitro experimental verification. Through this study, we identified the possible cardiovascular protection mechanism of SGLT2is in T2DM complicated with CHD, which provides a new perspective and research basis for cardiovascular protection and medical treatment of coronary heart disease. The overall flow of this study is shown in Figure 1.

## 2. Methods

### 2.1. Prediction of SGLT2is-related targets

Firstly, the chemical structures of 4 SGLT2is [canagliflozin (CANA), dapagliflozin (DAPA), empagliflozin (EMPA), and ertugliflozin (ERTU)] were obtained by PubChem (https://pubchem.ncbi.nlm.nih.gov), which is a publicly available chemical information resource database that stores a wide range of bioassay data from chemical genomics, medicinal chemistry, and functional genomics, including drug chemical structure.^[19,20]^ Then, in order to clarify the targets of drug action, we used the Swiss Target Prediction online tool (www.swisstargetprediction.ch) to map the 2D and 3D chemical structures of 4 SGLT2is and predict their potential targets.^[21]^ In addition, more SGLT2is-related target genes were obtained from DrugBank (https://go.drugbank.com/) and used in this study.^[22]^

### 2.2. Data collection on diabetes and coronary heart disease

Gene Expression Omnibus (https://www.ncbi.nlm.nih.gov/geo/, GEO) is a public database under the U.S. National Library of Medicine (https://www.nlm.nih.gov/, NLM) that collects genomic data on a wide range of diseases worldwide.^[23]^ In the GEO database, with “diabetes mellitus” and “coronary heart disease” as search terms, respectively, and the species as “Homo sapiens,” screening the matched datasets. Detailed inclusion criteria were: Experiment type was microarray or high-throughput sequencing; Provide raw RNA expression profiles; Provide detailed annotation information of RNA. Sequencing datasets with “other diseases in combination” and “healthy control samples missing from the experimental design” were not included in the selection.

### 2.3. Identification of differentially expressed genes in datasets

Differentially expressed genes (DEGs) of the datasets were analyzed using GEO2R. GEO2R (https://www.ncbi.nlm.nih.gov/geo/geo2r/) is an online analysis tool for GEO data based on the R programming language.^[24]^ *P* <.05 and |Log_2_FC|>1 were set as screening criteria, and genes in the dataset that met the criteria were considered to be differentially expressed.

### 2.4. Construct the “drug-target-disease (D-T-D)” network

To investigate the presence of SGLT2is targets in the disease DEGs and their specific functions, we explored the common genes between the SGLT2is targets and the 2 disease DEGs. The predicted targets of the 4 SGLT2is were taken as intersections with the DEGs of diabetes and CHD, and then the intersection results were visualized using Cytoscape (v3.4.0, www.Cytoscape.org) to construct the “D-T-D” relationship network.^[25]^

### 2.5. Functional enrichment analysis of target genes

Metascape (v 3.5, https://metascape.org) is an open resource for gene annotation and analysis, capable of functional enrichment analysis of genes, including gene ontology (GO) [including biological processes (BP), molecular functions (MF), and cellular components (CC)] and KEGG pathway.^[26]^ By importing target genes into Metascape and setting “Min Overlap = 3,” “*P*-value Cutoff <.05” and “Min Enrichment = 1.5” as the screening criteria, GO and KEGG enrichment analysis were performed on the target gene. String plots were used for visualization of GO results and Sankey bubble plots were used for presentation of KEGG results.

### 2.6. Constructing the PPI network of core targets

To understand whether there are associations between the target genes, we performed “protein-protein interaction (PPI)” analyses using STRING.^[27]^ STRING (https://cn.string-db.org/) is a web-based database based on public databases and literature information, which can provide the interaction relationship between gene-encoded proteins. The target gene name inputted into the “Multiple proteins” module and the species was set as “Homo sapiens” and the confidence level as >0.90 to obtain the interaction network of target gene proteins. In the PPI network results, nodes represent proteins and edges represent interactions between proteins. Cytoscape software was used to visualize the PPI network.

### 2.7. Screening of hub targets of SGLT2is in treatment of T2DM with CHD

The PPI network results were imported into Cytoscape software to study the topological features of the PPI network and the cytoHubba plugin was used for hub gene screening.^[28]^ A total of 6 algorithms (MCC, MNC, Degree, EPC, EcCentricity and Radiality) from the cytoHubba plugin were used in the hub gene identification process. These 6 algorithms are able to identify the nodes in the PPI network and rank each node to finally obtain the hub genes with high score ranking in the PPI network.

### 2.8. Molecular docking

First, the chemical structures of drug molecules and target proteins were obtained. The 2D structures of the 4 SGLT2is drug molecules [canagliflozin (CANA), dapagliflozin (DAPA), empagliflozin (EMPA), and ertugliflozin (ERTU)] were downloaded from the PubChem (https://pubchem.ncbi.nlm.nih.gov/) database, and the 3D structures of the target proteins (EGFR, ITGB2 and LCK) were downloaded from the RCSB Protein Data Bank (RCSB PDB, https://www.rcsb.org/) database. Then, the preprocessing of the molecules was performed. The PyMOL software was used to “remove solvent” and “remove organic” from the target proteins, Chem3D software was used to set the minimum free energy for the drug molecules and converts them into 3D structures, and the AutoDockTools software to perform the hydrogenation of the protein and drug molecule. Finally, the AutoDockTools software was used to dock the drug molecules to the target proteins, including setting up the Grid Box, running AutoGrid, running “AutoDock” and other steps. The drug molecule-target protein (D-T) docking results (binding energies) are visualized in the form of heatmaps, and the lower binding energy docking results for each core target are shown in detail by PyMOL software.

### 2.9. In vitro experiments

#### 2.9.1. H9c2 cardiomyocyte culture and the construction of a high-glucose and high fat-induced hypoxia-reoxygenation cardiomyocyte model

Rat H9c2 cells were used for cellular studies of myocardial injury. H9c2 cardiomyocytes were cultured in Dulbecco Modified Eagle Medium (DMEM) containing 10% FBS set at 37°C in normoxia (5% CO_2_ by volume). After 80% confluence, the cells were treated with high glucose (glucose 25 mmol/L) (HG) and high-lipid (palmitic acid 300 μmol/L) (HP) for 24 hours. The medium was then replaced with sugar-free serum-free medium, and the cells were hypoxic for 4 hours in a triple-gas incubator (94% N_2_, 5% CO_2_, and 1% O_2_), and then the cells were placed in the normoxic incubator to reoxygenate the cells for 2h.

H9c2 cells were divided into 6 groups: normal control group (NC, H9c2 cells cultured normally); normal control group treated with 2.5 μM of ERTU; normal controls treated with 5.0 μM of ERTU treatment group; high-glucose/high-lipid/+H/R treatment group (HG/HP + H/R); HG/HP + H/R with 2.5 μM ERTU treatment group; HG/HP + H/R with 5.0 μM ERTU treatment group.

#### 2.9.2. Enzyme-linked immunosorbent assay

H9c2 cells were inoculated in 6-well plates at a density of 1 × 10^5^ cells/well. Supernatants were taken after ERTU drug intervention, and enzyme markers were used to measure the absorbance at 450 nm of epidermal growth factor receptor (EGFR, ab126421, Abcam, China).

#### 2.9.3. Real-time quantitative PCR

Total RNA was isolated from H9c2 cells using the RNeasy mini kit according to the operation manual. The Revert Ace kit (TOYOBO, Japan) is used to reverse transcribe RNA samples into cDNA and then measure mRNA using SYBR-Green RT-qPCR. β-actin is used for the quantification of cDNA of EGFR. The primer sequences for EGFR is as follows: 5’-GGCCTCTTCTGCGATTTCG-3’(forward), 5’-GCAGCTTGACCCTTCTCGG-3’(reverse); β-actin: 5’-ATGGAGGGGAATACAGCCC-3’(forward), 5’-TTCTTTGCAGCTCCTTCGTT-3’(reverse).

#### 2.9.4. Western-blot analysis

SDS polyacrylamide gel electrophoresis (SDS-PAGE) was used to chemically separate protein lysates according to gel for wet transfer printing, and transfer buffer was prepared. PVDF transfer film and dry film were prepared, and the PVDF film was activated with anhydrous methanol for 30 seconds, rinsed with deionized water to remove organic solvents, and then equilibrated in transfer buffer for 5 minutes. The membrane was stirred and incubated with sufficient blocking buffer at room temperature for 30 to 60 minutes. After diluting the primary antibody in a closed buffer, the reaction was allowed to proceed overnight and then incubated with the corresponding secondary antibody. Finally, ELC was used to excite chemiluminescence and observe the expression of the target protein.

## 3. Statistical analysis

SPSS 22.0 (SPSS Inc., Chicago) and GraphPad Prism 8.3 software (GraphPad Software, San Diego) were used for data analysis, and Student *t* test (two-group comparison) and 1-way analysis of variance (ANOVA, 2 or more groups) were used for intergroup comparison. All data are presented as the mean ± standard error of the mean (SEM), and statistical charts are plotted based on the statistical results. *P* <.05 is considered statistically significant.

## 4. Results

### 4.1. Obtain potential targets for SGLT2is

The molecular structures and Canonical SMILES of the 4 SGLT2is [canagliflozin (CANA), dapagliflozin (DAPA), empagliflozin (EMPA), and ertugliflozin (ERTU)] can be obtained from PubChem. The details of their structure are shown in Table 1. The 4 SGLT2 inhibitors share a common parent nucleus based on their chemical structures, although having somewhat differing side-chain substituents.

**Table 1:**
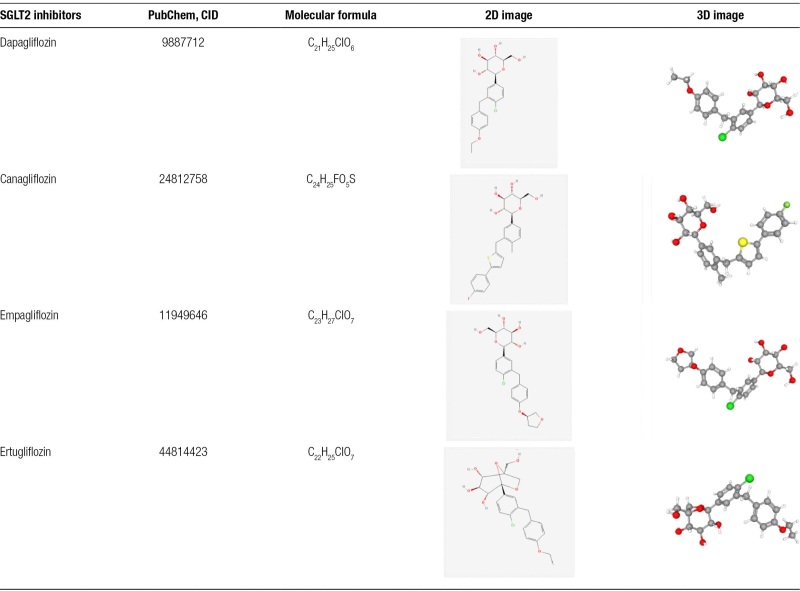
Detailed information and molecular structures of 4 SGLT2 inhibitors.

**Figure 2.**
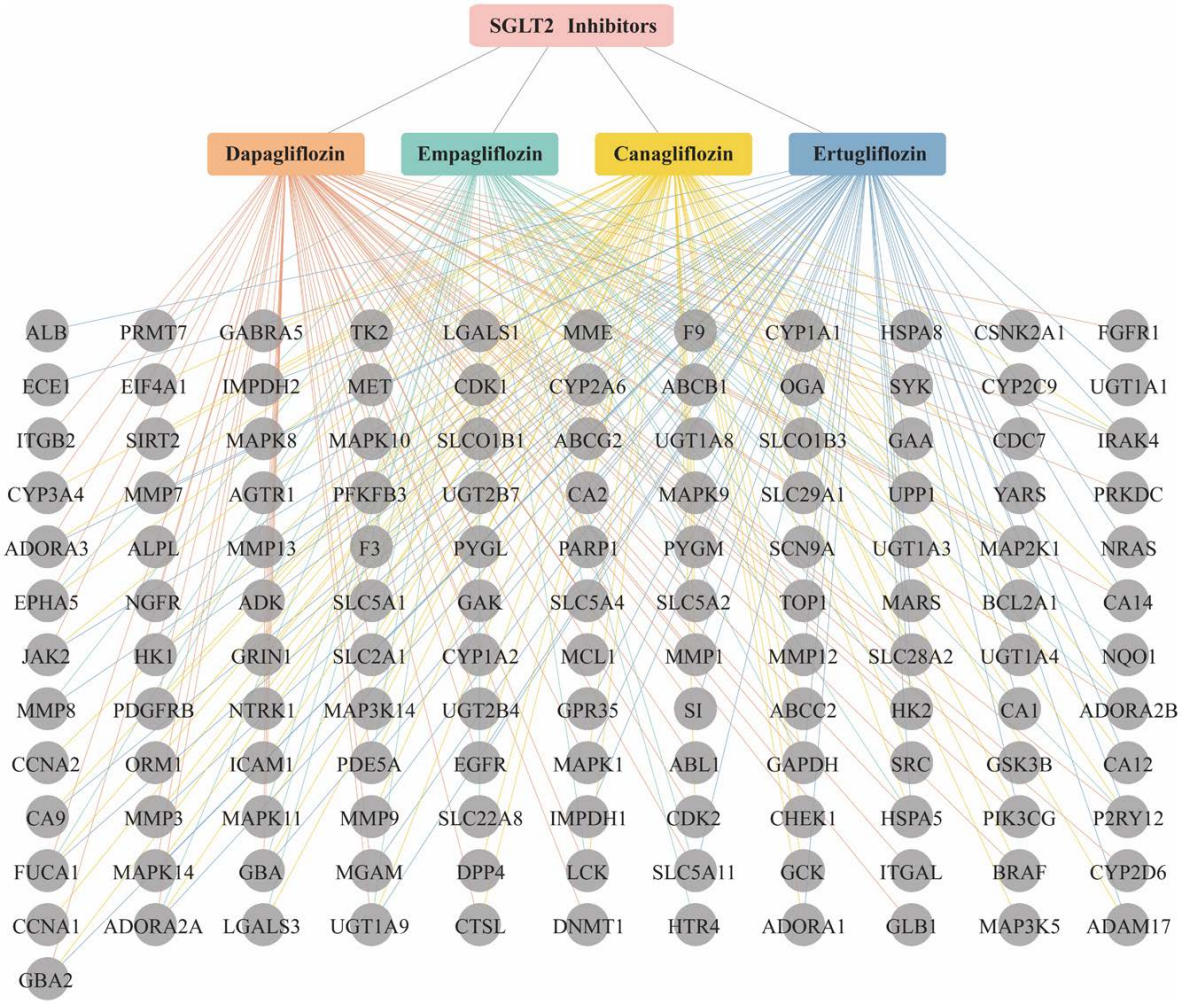
Target prediction of 4 SGLT2is. Interaction diagram of 4 SGLT2 inhibitors with their targets. There were 137 nodes (including 4 drugs, 133 predicted targets) and 283 edges, Pink rectangle represents 4 types of SGLT2is (orange rectangle represents dapagliflozin, green rectangle represents empagliflozin, yellow rectangle represents canagliflozin and blue rectangle represents ertugliflozin), gray dots represents the predicted target of SGLT2is, and 1 SGLT2i can be connected with multiple targets, representing the interaction between 1 drug and multiple targets. SGLT2is = sodium-glucose co-transporter 2 inhibitors.

Potential targets for the 4 SGLT2is were obtained from SwissTargetPrediction and Drugbank databases. By importing the molecular structures of the 4 SGLT2is into the SwissTargetPrediction database, 242 targets with a probability >0 were screened, including 70 targets of dagliflozin, 69 targets of cagliflozin, 60 targets of ertugliflozin, and 43 targets of empagliflozin. In the DrugBank database, we obtained 37 targets for 4 SGLT2 inhibitors, including 11 targets for dagliflozin, 10 targets for empagliflozin, 8 targets for canagliflozin, and 8 targets for ertugliflozin. Thus, we obtained a total of 279 targets for the 4 SGLT2i drugs. Then after integration and duplicate elimination of 279 data, a total of 133 independent targets for the 4 SGLT2i drugs were obtained. The 133 targets of the 4 SGLT2is and their matching relationships are presented in Figure 2.

### 4.2. Acquisition of DEGs in coronary heart disease and type 2 diabetes mellitus

**Figure 3.**
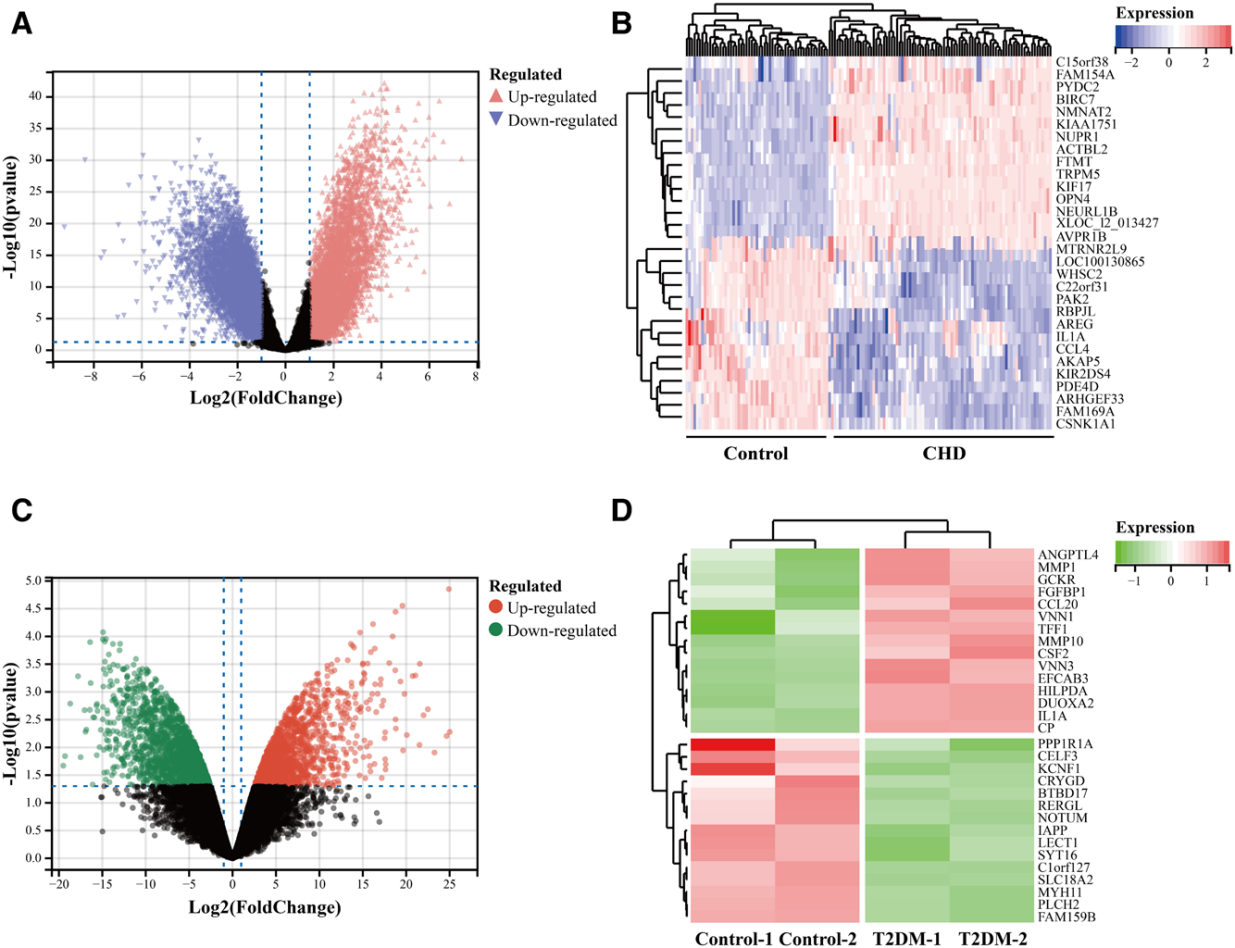
DEGs analysis of coronary heart disease dataset GSE113079 and diabetes mellitus dataset GSE118139. (A) Volcano plot of gene expression distribution in coronary heart disease dataset GSE113079. *P* <.05 and |Log_2_FC|>1 were set as the screening criteria, pink triangles represent up-regulated genes and purple inverted triangles represent down-regulated expressed genes, and black dots indicate meaningless genes. (B) Heatmap of the top 30 DEGs in the coronary heart disease dataset GSE113079. Red color indicates up-regulation of gene expression and purple color indicates down-regulation of gene expression. (C) Volcano plot of gene expression distribution in the diabetes dataset GSE118139. Setting *P* <.05 and |Log_2_FC|>1 as the screening criteria, red circles represent up-regulated genes, green circles represent down-regulated expressed genes, and black dots represent meaningless genes. (D) Heatmap of the top 30 DEGs for the diabetes dataset GSE118139. Red color indicates up-regulation of gene expression and green color indicates down-regulation of gene expression. DEGs = differentially expressed genes.

From the GEO database, we retrieved the coronary heart disease dataset GSE113079 and the diabetes dataset GSE118139 with human sample sources. The GSE113079 dataset collected mRNA data from peripheral blood mononuclear cells (PBMC) of 93 patients with CHD and 48 healthy controls; the GSE118139 dataset collected mRNA data from 2 T2DM patients and 2 nondiabetic patients with islet genes. By performing statistical analysis of the GSE113079 and GSE118139 datasets to obtain DEGs associated with CHD and T2DM. By setting the conditions of *P* <.05 and |Log_2_FC|>1, 10,240 DEGs were identified in GSE113079 dataset, including 5348 up-regulated genes and 4892 down-regulated genes. The volcano map (Fig. 3A) shows the overall gene distribution of the dataset GSE113079, while the heatmap (Fig. 3B) displays the inter-sample expression of the top 30 DEGs. In the GSE118139 dataset, 3398 DEGs were identified, including 1441 up-regulated genes and 1957 down-regulated genes (Fig. 3C). Figure 3D shows the inter-sample expression of the top 30 DEGs. Details of the 2 datasets are shown in Table 2, including the source of the organization and the sample size.

**Table 2:** Details of the coronary heart disease and diabetes dataset

GEO accession	Experiment type	Species	Disease model	Source tissue	Sample		Data
					Control	Disease	
GSE113079	Noncoding RNA profiling by array	Homo sapiens	CAD	peripheral blood mononuclear cells	48	93	mRNA
GSE118139	Expression profiling by array	Homo sapiens	T2DM	Islets	2	2	mRNA

CAD = coronary artery disease, T2DM = type 2 diabetes mellitus.

### 4.3. Acquisition the co-acting targets of SGLT2is in T2DM and CHD

**Figure 4.**
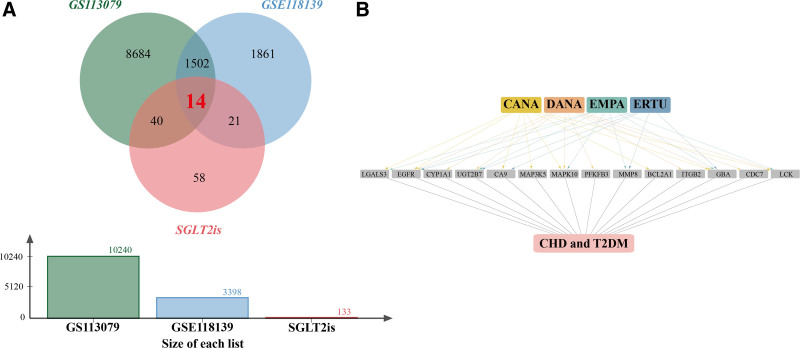
Drug-target-disease network of SGLT2 inhibitors. (A) Venn plots of the intersection of SGLT2i drug targets with DEGs in the CHD and DM datasets. Green color represents 10,240 DEGs from the CHD dataset, blue color represents 3398 DEGs from the T2DM dataset, and pink color represents 133 predicted targets for the 4 SGLT2is. The intersection of the 3 yielded 14 common genes. (B) Drug-target-disease network of SGLT2 inhibitors. The network consists of 20 nodes and 43 edges. Orange dots represent dapagliflozin, green dots represent empagliflozin, yellow dots represent canagliflozin, blue dots represent ertugliflozin, gray dots represent common targets of drugs and diseases, and pink dots represent CHD and T2DM. CHD = coronary heart disease, DEGs = differentially expressed genes, DM = diabetes mellitus, SGLT2is = sodium-glucose co-transporter 2 inhibitors, T2DM = type 2 diabetes mellitus.

After completing the analysis of DEGs in the T2DM and CHD datasets, we further overlapped the 2 datasets. The results showed that a total of 1516 DEGs were co-occurring in dataset GSE113079 and dataset GSE118139, suggesting that these genes are shared targets in diabetes and CHD disease progression (Fig. 4A). Then, to investigate the potential targets of SGLT2i in T2DM and CHD, we crossed the 1516 shared DEGs with 133 drug targets and finally obtained 14 SGLT2i drug targets that were differentially expressed in both T2DM and CHD (Fig. 4A). Figure 4B demonstrates the relationship between the 14 target genes and the 4 SGLT2i drugs, T2DM and CHD.

### 4.4. GO and KEGG enrichment analysis of core targets

**Figure 5.**
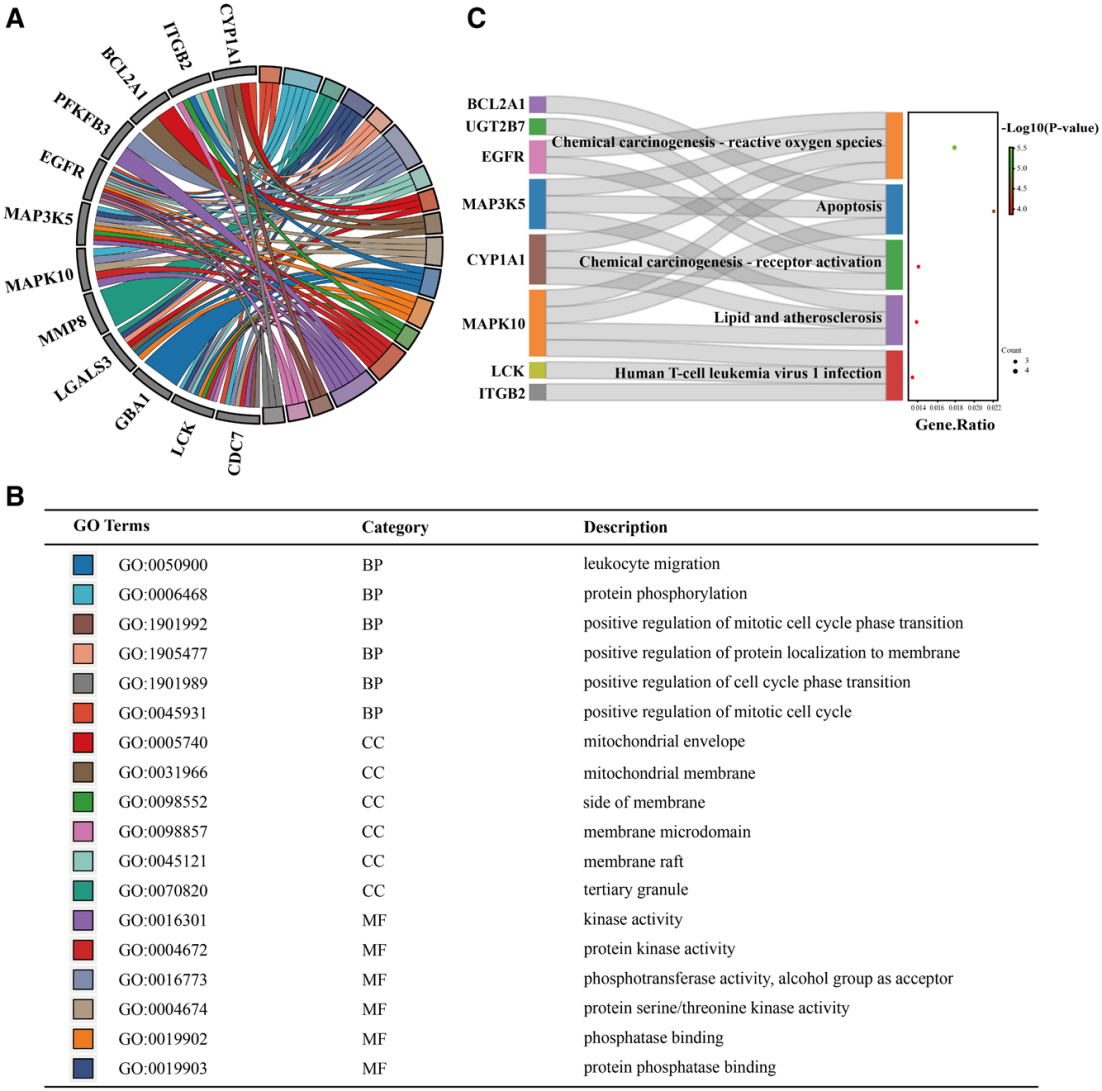
Functional enrichment analysis of GO/KEGG. (A) Chordal plots of the results of GO enrichment analysis of 14 target genes. (B) Presentation of the first 15 terms of GO enrichment analysis results. (C) Sankey bubble plots of the top 5 pathways from KEGG pathway analysis results for 14 target genes. GO = gene ontology, KEGG = Kyoto encyclopedia of genes and genomes.

In order to more systematically investigate the regulatory mechanisms of target genes in T2DM and coronary artery disease, we performed GO/KEGG analyses on 14 shared targets. Metascape analysis showed that leukocyte migration, protein phosphorylation, and positive regulation of mitotic cell cycle phase transition were the top 3 biological processes enriched for target genes, the tertiary granule, membrane raft and membrane microdomain were the top 3 enriched cellular components, and phosphotransferase activity, alcohol group as acceptor, kinase activity and protein phosphatase binding were the top 3 enriched molecular functions (Fig. 5A and B). KEGG pathway enrichment analysis showed that chemical carcinogenesis-reactive oxygen species, apoptosis and chemical carcinogenesis-receptor activation were the major signaling pathways involved in 14 target genes (Fig. 5C). Chordal and Sankey bubble plots demonstrate the major GO/KEGG entries enriched for the 14 target genes.

### 4.5. PPI network and analysis of core target

**Figure 6.**
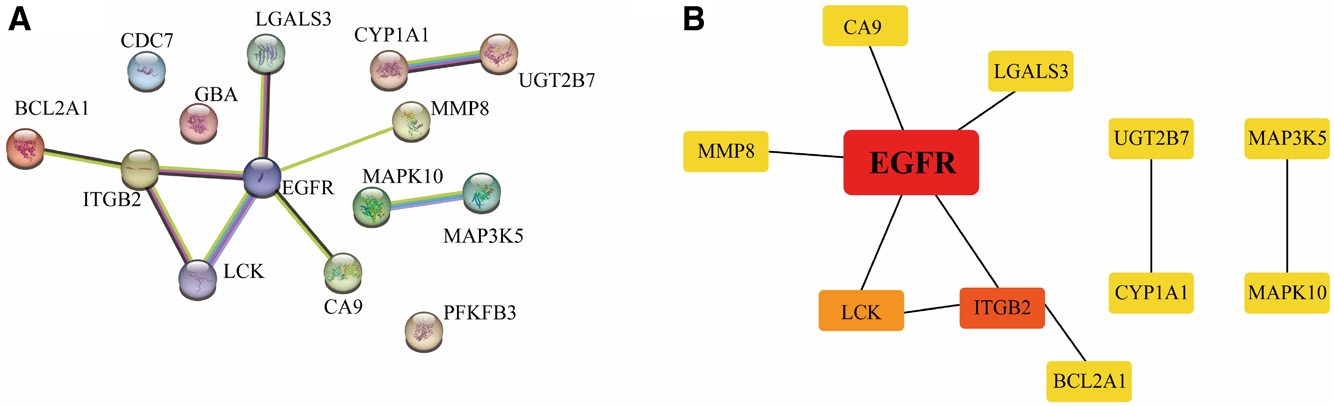
PPI network and hub gene network of 14 target genes. (A) PPI network for 14 SGLT2is targets. Each circle represents a protein, and the center of the circle represents the protein structure of the target gene. The line between the 2 points represents the interaction between proteins. (B) Hub gene network. A total of 11 hub genes of the PPI network were screened according to the 6 algorithms of the cytoHubba plugin. The redder the color of the dot, the more central the gene is. PPI = protein–protein interaction, SGLT2is = sodium-glucose co-transporter 2 inhibitors.

After clarifying the biological functions played by the target genes and their involvement in signaling pathways, we explored the hub genes among the 14 target genes. The results of the PPI network showed that only 11 of the 14 target genes interacted with each other at the protein level, and the other 3 target genes’ related proteins existed independently and did not crosstalk with other proteins (Fig. 6A). Then, in order to clarify the network topology of these 11 interrelated target genes, we performed hub gene analysis on the PPI network. Cytohubba analysis of the PPI network showed that epidermal growth factor receptor (EGFR) had the highest score in all 6 algorithmic results, followed by LCK proto-oncogene, src family tyrosine kinase (LCK) and integrin subunit beta-2 (ITGB2). This implies that EGFR may be a key target for the action of SGLT2 inhibitors as a hub gene for the treatment of diabetes and CHD; meanwhile, ITGB2 and LCK are important for the SGLT2 inhibitors for the treatment of CHD combined with diabetes are also critical. Table 3 shows the detailed scores and rankings of the 5 algorithms for the 11 target genes in the network. The top 11 genes in the PPI network are visualized in Figure 6B.

**Table 3:** Hub gene screening of cytohubba and results of 6 algorithms.

Gene name	Protein name	MCC	MNC	Degree	EPC	EcCentricity	Radiality
MAPK10	mitogen-activated protein kinase 10	1	1	1	1.482	0.18182	0.54545
MAP3K5	mitogen-activated protein kinase kinase kinase 5	1	1	1	1.482	0.18182	0.54545
LCK	LCK proto-oncogene, Src family tyrosine kinase	2	2	2	3.563	0.31818	1.90909
LGALS3	Galectin 3	1	1	1	2.771	0.21212	1.69697
MMP8	Matrix metallopeptidase 8	1	1	1	2.84	0.21212	1.69697
UGT2B7	UDP-glucuronosyltransferase 2B7	1	1	1	1.488	0.18182	0.54545
CYP1A1	Cytochrome P450 1A1	1	1	1	1.488	0.18182	0.54545
EGFR	Epidermal growth factor receptor	5	2	5	4.116	0.31818	2.22727
CA9	Carbonic anhydrase 9	1	1	1	2.885	0.21212	1.69697
ITGB2	Integrin beta-2	3	2	3	3.683	0.31818	2.01515
BCL2A1	Bcl-2-related protein A1	1	1	1	2.696	0.21212	1.48485

EGFR = epidermal growth factor receptor, ITGB2 = integrin subunit beta-2, LCK = LCK proto-oncogene, Src family tyrosine kinase

### 4.6. Analysis of molecular docking results

**Figure 7.**
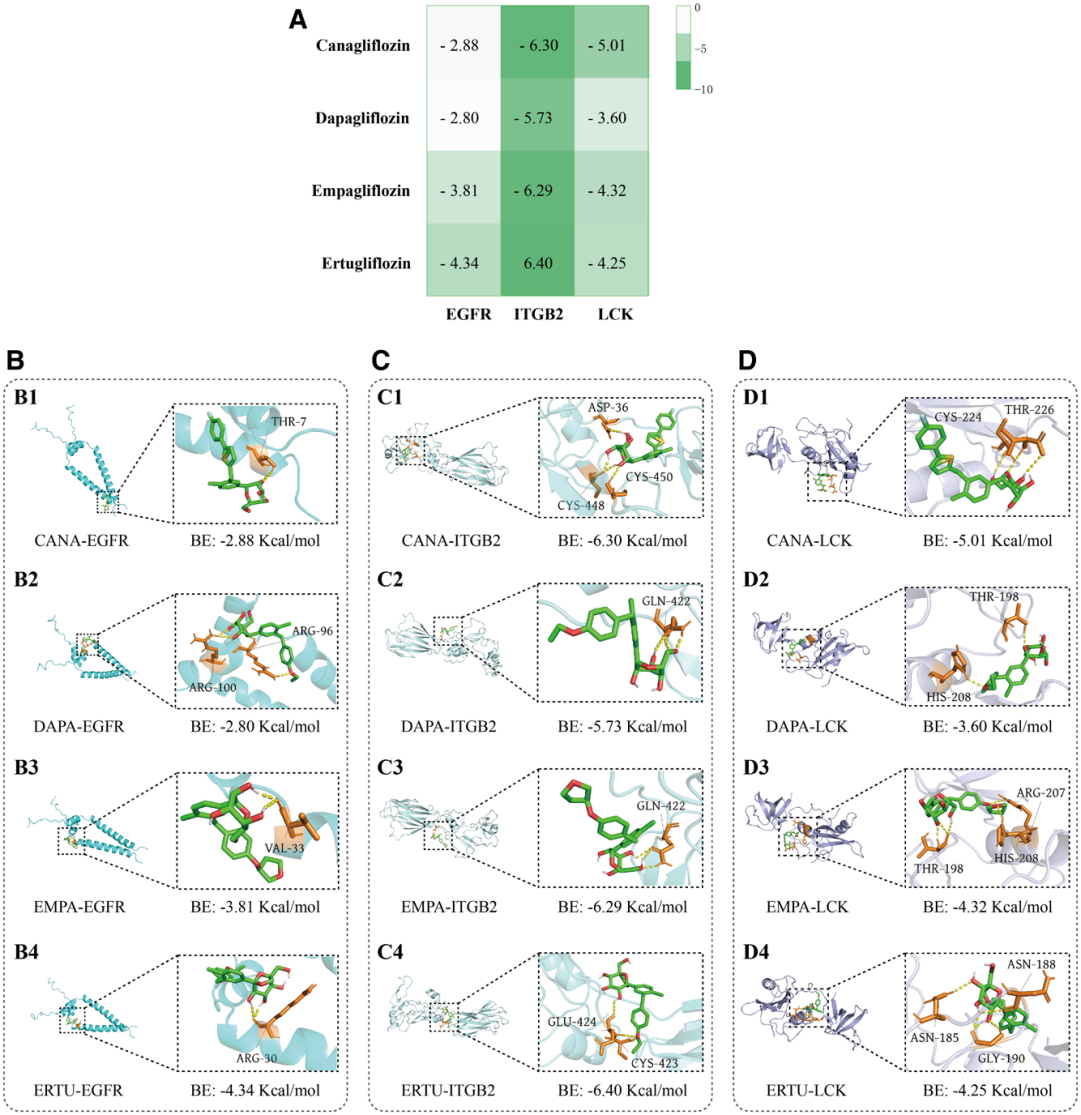
AutoDockTools molecular docking. (A) Molecular docking binding energy heatmap. The lower the binding energy, the stronger the intermolecular binding. (B) 3D binding patterns of 4 types of SGLT2is with EGFR. Green small molecules represent various SGLT2i drugs, while yellow represents intermolecular hydrogen bonds. (C) 3D binding patterns of 4 types of SGLT2is with ITGB2. (D) 3D binding patterns of 4 types of SGLT2is with LCK. ARG = arginine, ASN = asparagine, ASP = aspartate, BE = binding energy, CYS = cysteine, EGFR = epidermal growth factor receptor, GLN = glutamine, GLU = glutamate, GLY = glycine, HIS = histidine, ITGB2 = integrin subunit beta-2, LCK = LCK proto-oncogene, src family tyrosine kinase, SGLT2is = Sodium-glucose co-transporter 2 inhibitors, THR = threonine, VAL = valine.

To verify the reliability of the network pharmacology results, we conducted molecular docking between the top 3 core targets (EGFR, ITGB2, and LCK) and 4 SGLT2is (CANA, DAPA, EMPA, and ERTU) to evaluate the binding performance between molecules. It is generally believed that a binding energy of less than −1.2Kcal/mol represents a valid result. The molecular docking results showed that all 4 SGLT2is had binding energies less than −1.2Kcal/mol to the core target. The heatmap shows the binding energies of 4 SGLT2is molecules to target proteins, ranging from −6.4 to −2.80 Kcal/mol, with ERTU exhibiting the strongest affinity for ITGB2 (-6.40 Kcal/mol) (Fig. 7A). Notably, all 4 SGLT2is had lower levels of binding energies to ITGB2 proteins, and comparatively, CANA and DAPA demonstrated a weaker ability to bind to EGFR proteins, at −2.88 and −2.80 Kcal/mol, respectively.

After determining the optimal binding state between each drug molecule and the target protein, we further visualized the binding sites between the drug molecule and the target protein. As shown in Figure 7C-C4, ERTU interacted with ITGB2 by forming 2 hydrogen bonds with GLU-424 and CYS-423 active sites; EMPA was linked to amino acid residue VAL-33 on EGFR protein through 2 hydrogen bonds (Fig. 7B-B3). In LCK protein binding, EMPA formed 5 hydrogen bonds with 3 amino acid residues THR-198, HIS-208, and ARG-207, forming a stable binding with −4.32 Kcal/mol binding energy (Fig. 7D-D3). Figure 7B-D shows in detail the 3D binding patterns of the 4 SGLT2is drug molecules to the active sites of the target proteins and highlights the key amino acid residues and hydrogen bonds. These results indicate strong binding between SGLT2is drug molecule-target and suggest that the target protein is in an optimal conformation.

### 4.7. SGLT2i-ertugliflozin enhances HG/HP + H/R-induced H9c2 cardiomyocyte viability

**Figure 8.**
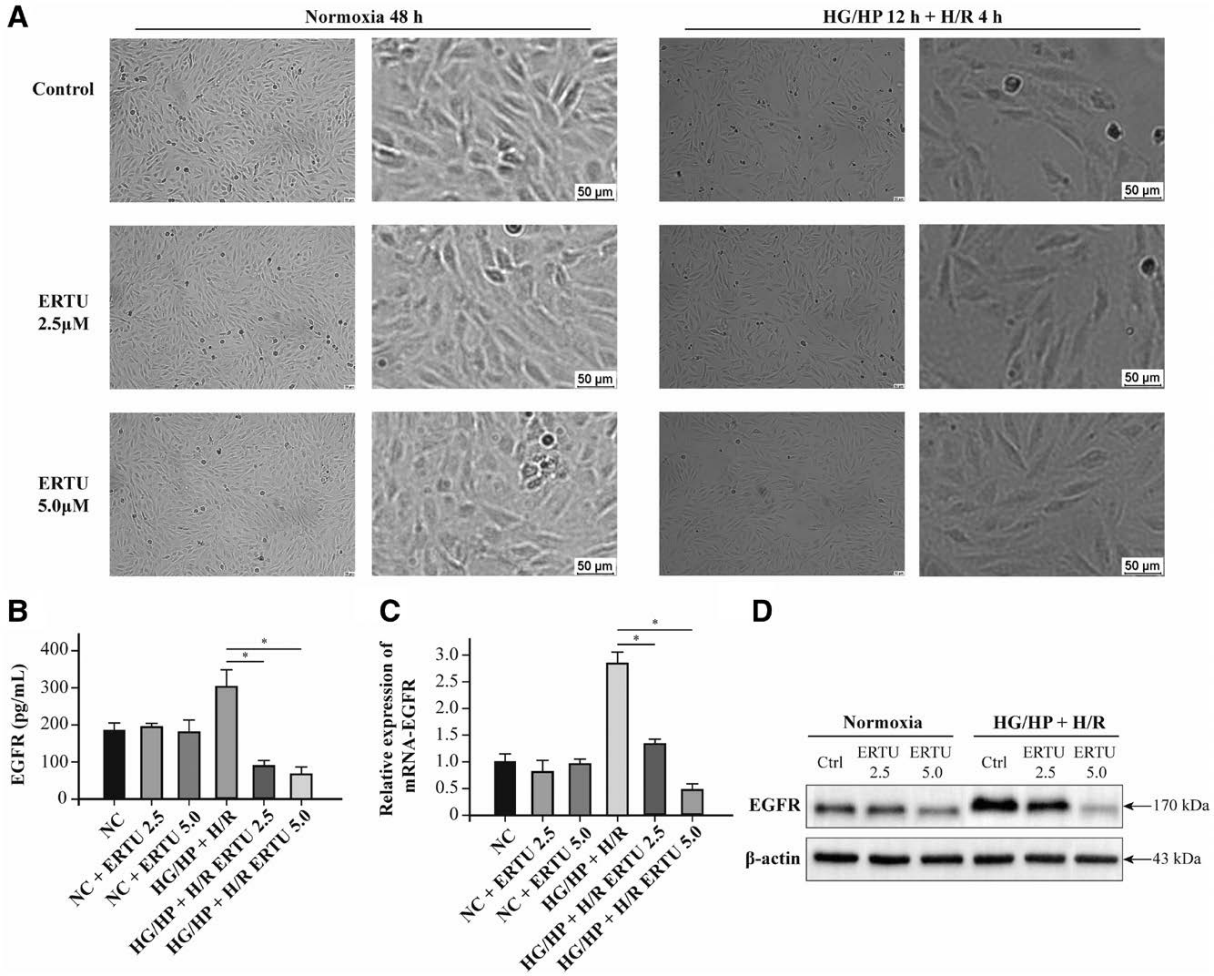
H9c2 cell experiment verifies that SGLT2i-ERTU improves HG/HP + H/R myocardial cell injury. (A) Appearance of H9c2 cardiomyocytes under normoxic, HG/HP + H/R culture, and ERTU treatment. (B) ELISA was used to measure the EGFR content in the supernatant of H9c2 cell culture. *P <.05. (C) Histogram of relative expression of EGFR mRNA in H9c2 cardiomyocytes induced by normoxicity and HG/HP + H/R combined with ERTU treatment. *P <.05. (D) Western blotting analysis was used to detect the protein expression of EGFR in H9c2 cardiomyocytes induced by normoxic and HG/HP + H/R with ERTU treatment. ELISA = enzyme-linked immunosorbent assay, ERTU = ertugliflozin, HG/HP + H/R = high-glucose/high-lipid combined hypoxia-reoxygenation, SGLT2i = sodium-glucose co-transporter 2 inhibitor.

The results of cell culture and intervention showed that the presence of SGLT2i-ERTU had no significant regulatory effect on the proliferation of H9c2 cardiomyocytes under normoxic conditions (Fig. 8A) shows that under normoxic conditions, the presence of SGLT2i-ERTU showed no significant modifying effect on the proliferation of H9c2 cardiomyocytes. Consistent with the expected results, H9c2 cardiomyocytes showed a significant decrease in cell viability after 4 hours of HG/HP + H/R incubation. Interestingly, 2.5 μM and 5.0 μM ERTU interventions effectively reversed the reduction in cell proliferation viability induced by HG/HP + H/R (Fig. 8A).

### 4.8. SGLT2i-ertugliflozin inhibits EGFR expression in HG/HP + H/R cardiomyocytes

We further used ELISA to detect EGFR levels in cardiomyocyte culture supernatants. As shown in Figure 8B, EGFR levels were significantly reduced after 2.5 μM and 5.0 μM ERTU treatment (*P *<.05) compared with HG/HP + H/R-treated H9c2 cells. At the mRNA level, EGFR expression also showed a consistent trend (Fig. 8C). To clarify the changes of EGFR at the protein level, we performed western-blot experiments. The results showed that ERTU significantly inhibited EGFR protein expression in cardiomyocytes of the HG/HP + H/R group (*P* <.05) (Fig. 8D). This suggests that EGFR is a downstream target of SGLT2i, which acts on EGFR to ameliorate myocardial injury caused by HG/HP H/R.

## 5. Discussion

Coronary heart disease is the leading cause of death in patients with type 2 diabetes mellitus. Therefore, it is very important to prevent the occurrence and damage of CHD in patients with type 2 diabetes mellitus to improve the prognosis of patients. Existing clinical studies suggest that SGLT2is also have a favorable protective effect on cardiac function in patients with diabetes mellitus combined with CHD, but the mechanism between them is unclear.^[29]^ Therefore, this study integrated network pharmacology and bioinformatics analysis to explore the specific mechanism and therapeutic targets of SGLT2is to improve myocardial injury in patients with T2DM and CHD.

Through drug-target prediction and DEGs analysis of the dataset, we screened 14 common target genes from the drug targets of SGLT2is and the DEGs of 2 diseases (CHD and T2DM), and then constructed the “drug-target-disease (D-T-D)” network and PPI network. We performed GO and KEGG analyses on the core targets and found that the mechanism by which SGLT2is regulate the progression of diabetes mellitus complicated with coronary heart disease may involve the following GO terms, such as leukocyte migration, the tertiary granule, and phosphotransferase activity, alcohol group as acceptor. In the KEGG pathway, chemical carcinogenesis-reactive oxygen species, apoptosis and chemical carcinogenesis-receptor activation are the major signaling pathways involved in the 14 target genes. Then, according to cytoHubba’s 6 algorithms, we identified the hub gene EGFR in the PPI network, as well as key genes such as ITGB2 and LCK, which may be important genes for SGLT2i treatment of diabetic coronary heart disease. This demonstrates the multi-target and multi-pathway characteristics of SGLT2i in the treatment of coronary heart disease combined with diabetes therapy.

Based on the results of GO functional enrichment analysis, we found that these core targets are focused on biological processes such as leukocyte migration, protein phosphorylation, and positive regulation of mitotic cell cycle phase transition. Studies have shown that excess leukocyte transendothelial migration and endothelial activation are important factors in accelerating the development of atherosclerotic plaques.^[30]^ Leukocyte adherence to endothelial cells is facilitated by C-reactive protein (CRP), intercellular adhesion molecule-1 (ICAM-1), and vascular cell adhesion molecule-1,^[31,32]^ stimulates the migration and proliferation of vascular smooth muscle cells within the neointima, resulting in luminal blockage, leading to atherosclerosis. At the same time, recent studies have also shown that reduced leukocyte migration is associated with a reduced incidence of atherosclerosis and more durable atherosclerotic plaques.^[33]^

KEGG pathway enrichment analysis showed that chemical carcinogenesis-reactive oxygen species, apoptosis and chemical carcinogenesis-receptor activation were the major signaling pathways. Apoptosis is a coordinated and self-killing process and is an important risk factor for the development of coronary artery disease.^[34,35]^ Abbate et al showed that excessive cardiomyocyte apoptosis can promote left ventricular remodeling.^[36]^ Nilsson et al and Abbate et al showed that increased apoptosis is observed in patients with acute and subacute myocardial ischemia.^[37,38]^ The results suggest that SGLT2is may regulate the progression of diabetes mellitus and coronary heart disease by acting on target genes to regulate biological processes such as protein phosphorylation and leukocyte transcortical migration, as well as pathways such as apoptosis.

Molecular docking uses computational simulation techniques to obtain the optimal binding conformation between ligands and receptors (L-R), and provides binding energy results.^[39]^ The lower the binding energy between drug ligands and protein receptors, the higher the possibility of intermolecular binding. Moreover, intermolecular hydrogen bonds play a crucial role in maintaining the stability of L-R complexes.^[40]^ In our docking results, the binding energies of the 12 best molecular docking results were all less than −1.2Kcal/mol, indicating stable binding between SGLT2is and EGFR, ITGB2, and LCK targets. In EGFR target proteins, ERTU forms 2 hydrogen bonds with Arg-30 and generates a binding energy of −4.34 kcal/mol, suggesting its potential as an effective kinase inhibitor; Moreover, Liao et al proposed that the methylation of Arg198 and Arg200 residues in the extracellular domain of EGFR positively regulates the binding affinity between EGF and EGFR.^[41]^ This provides a basis for the study of EGFR modification in the improvement of myocardial injury in T2DM complicated with CHD.

Based on network pharmacology analysis and molecular docking results, SGLT2is have been shown to have strong binding affinity with target proteins EGFR, ITGB2, and LCK. However, these results still require further experimental validation. By tracing the expression changes of EGFR, ITGB2 and LCK in the dataset GSE113079 and GSE118139, we found that among the 3 target genes, only EGFR showed the same change trend in CHD and T2DM, while the expression trend of ITGB2 and LCK was reversed. We collated the fold changes of EGFR, ITGB2, and LCK in the dataset GSE113079 and GSE118139, and generated histograms to show their respective trends (Fig. S1, Supplemental Digital Content, https://links.lww.com/MD/R214). At the same time, the molecular docking results showed that ertugliflozin had strong molecular binding to EGFR. Therefore, we used ertugliflozin as the drug representative and EGFR as the core target to study the specific mechanism of SGLT2is in improving myocardial injury in CHD complicated with T2DM. We constructed a high-glucose/high-lipid hypoxia/reoxygenation (HG/HP + H/R) model using H9c2 cardiomyocytes to simulate the state of CHD with T2DM. The results showed that the proliferation ability of cardiomyocytes was significantly reduced in the HG/HP H/R state, while the ERTU of 2.5 μM and 5.0 μM could improve the viability of cardiomyocytes. In addition, 2.5 μM and 5.0 μM ERTU inhibited EGFR expression in cardiomyocytes in the HG/HP H/R state, which may be the key to improved cardiomyocyte viability.

The epidermal growth factor receptor (EGFR) is a member of the family of tyrosine kinase receptors on the cell surface. It is activated by binding to epidermal growth factor and plays an important regulatory role in cardiac fibrosis and remodeling.^[32,42]^ Bermadani et al found that high phosphorylation levels of EGFR can lead to increased arterial resistance in type 2 diabetes. Zhang et al concluded that inhibiting EGFR phosphorylation can slow down the progression of diabetes nephropathy by reducing endoplasmic reticulum stress and increasing autophagy.^[43]^ Zhang et al found that nephronectin (NPNT) promotes myocardial repair and improves cardiac function by activating the EGFR signal pathway to regulate angiogenesis and reduce myocardial fibrosis.^[44]^ All of these studies suggest that EGFR is an essential regulator in the protection of vascular function, including in patients with T2DM and CHD.

## 6. Conclusions

In conclusion, through bioinformatics and network pharmacology analysis, our study systematically analyzed the potential targets, pathways, and possible mechanisms of SGLT2is regulating the progression of T2DM with CHD, and validated the results using molecular docking and cellular experiments. The results suggest that EGFR may be a key target for the treatment of T2DM and CHD, and SGLT2i-ERTU can effectively inhibit EGFR expression and improve the viability of cardiomyocytes induced by HG/HP + H/R, which may be the key for SGLT2is to exert myocardial protection in T2DM combined with CHD. These findings provide solid data support and new experimental research directions for studying the pharmacological mechanism and disease treatment of SGLT2 inhibitors. However, it is important to note that the molecular docking model in this study does not take into account the flexibility of the protein, which may overestimate binding stability. In addition, this study failed to examine the downstream regulatory mechanisms of EGFR and cardiomyocyte function in more depth, and subsequent molecular dynamics simulations and in vitro experiments will help deepen the current research.

## Author contributions

**Conceptualization:** Shuai Yu, Jingru Li.

**Data curation:** Chaguo Li.

**Formal analysis:** Jingru Li.

**Funding acquisition:** Ping Yang, Luqiao Wang.

**Methodology:** Shuai Yu.

**Software:** Shuai Yu, Jingru Li.

**Supervision:** Hao Guo, Ping Yang, Luqiao Wang.

**Validation:** Xinyu Wu, Si Lu, Huan Cheng.

**Writing – original draft:** Shuai Yu, Jingru Li.

**Writing – review & editing:** Shuai Yu, Jingru Li, Huan Cheng, Ping Yang, Luqiao Wang.

## Supplementary Material



## References

[1] Zhang Y Zhang XO Chen T. Circular intronic long noncoding RNAs. Mol Cell. 2013;51:792–806. 24035497 10.1016/j.molcel.2013.08.017

[2] Huang L Wu P Zhang Y. Relationship between onset age of type 2 diabetes mellitus and vascular complications based on propensity score matching analysis. J Diabetes Investig. 2022;13:1062–72. 10.1111/jdi.13763 PMC9153842 35119212

[3] Low Wang CC Hess CN Hiatt WR Goldfine AB. Clinical update: cardiovascular disease in diabetes mellitus: atherosclerotic cardiovascular disease and heart failure in type 2 diabetes mellitus – mechanisms, management, and clinical considerations. Circulation. 2016;133:2459–502. 27297342 10.1161/CIRCULATIONAHA.116.022194 PMC4910510

[4] Megaly M Schmidt CW Dworak MW. Diabetic patients who present with ST-elevation myocardial infarction. Cardiovasc Revasc Med. 2022;38:89–93. 34373234 10.1016/j.carrev.2021.08.003

[5] Neuen BL Young T Heerspink HJL. SGLT2 inhibitors for the prevention of kidney failure in patients with type 2 diabetes: a systematic review and meta-analysis. Lancet Diabetes Endocrinol. 2019;7:845–54. 31495651 10.1016/S2213-8587(19)30256-6

[6] Tentolouris A Vlachakis P Tzeravini E Eleftheriadou I Tentolouris N. SGLT2 inhibitors: a review of their antidiabetic and cardioprotective effects. Int J Environ Res Public Health. 2019;16:2965. 31426529 10.3390/ijerph16162965 PMC6720282

[7] Scisciola L Cataldo V Taktaz F. Anti-inflammatory role of SGLT2 inhibitors as part of their anti-atherosclerotic activity: data from basic science and clinical trials. Front Cardiovasc Med. 2022;9:1008922. 36148061 10.3389/fcvm.2022.1008922 PMC9485634

[8] Terasaki M Hiromura M Mori Y. Amelioration of hyperglycemia with a sodium-glucose cotransporter 2 inhibitor prevents macrophage-driven atherosclerosis through macrophage foam cell formation suppression in type 1 and type 2 diabetic mice. PLoS One. 2015;10:e0143396. 26606676 10.1371/journal.pone.0143396 PMC4659635

[9] Vaduganathan M Januzzi JL Jr. Preventing and treating heart failure with sodium-glucose co-transporter 2 inhibitors. Am J Cardiol. 2019;124(Suppl 1):S20–7. 31741436 10.1016/j.amjcard.2019.10.026

[10] Li S Zhang B. Traditional Chinese medicine network pharmacology: theory, methodology and application. Chin J Nat Med. 2013;11:110–20. 23787177 10.1016/S1875-5364(13)60037-0

[11] Zhang R Zhu X Bai H Ning K. Network pharmacology databases for traditional Chinese medicine: review and assessment. Front Pharmacol. 2019;10:123. 30846939 10.3389/fphar.2019.00123 PMC6393382

[12] Wu CW Lu L Liang SW Chen C Wang SM. [Application of drug-target prediction technology in network pharmacology of traditional Chinese medicine]. Zhongguo Zhong Yao Za Zhi. 2016;41:377–82. 28868850 10.4268/cjcmm20160303

[13] Liu T Wang J Tong Y. Integrating network pharmacology and animal experimental validation to investigate the action mechanism of oleanolic acid in obesity. J Transl Med. 2024;22:86. 38246999 10.1186/s12967-023-04840-x PMC10802007

[14] Zhou HM Yue SJ Wang WX. Exploring the effective compounds and potential mechanisms of Shengxian Decoction against coronary heart disease by UPLC-Q-TOF/MS and network pharmacology analysis. Heliyon. 2024;10:e29558. 38681620 10.1016/j.heliyon.2024.e29558 PMC11046127

[15] Pinzi L Rastelli G. Molecular docking: shifting paradigms in drug discovery. Int J Mol Sci . 2019;20:4331. 31487867 10.3390/ijms20184331 PMC6769923

[16] Ferreira LG Dos Santos RN Oliva G Andricopulo AD. Molecular docking and structure-based drug design strategies. Molecules (Basel, Switzerland). 2015;20:13384–421. 26205061 10.3390/molecules200713384 PMC6332083

[17] Li X Wei S Niu S. Network pharmacology prediction and molecular docking-based strategy to explore the potential mechanism of Huanglian Jiedu decoction against sepsis. Comput Biol Med. 2022;144:105389. 35303581 10.1016/j.compbiomed.2022.105389

[18] Han J Hou J Liu Y Liu P Zhao T Wang X. Using network pharmacology to explore the mechanism of panax notoginseng in the treatment of myocardial fibrosis. J Diabetes Res. 2022;2022:8895950. 35372585 10.1155/2022/8895950 PMC8975676

[19] Wang Y Xiao J Suzek TO Zhang J Wang J Bryant SH. PubChem: a public information system for analyzing bioactivities of small molecules. Nucleic Acids Res. 2009;37:W623–633. 19498078 10.1093/nar/gkp456 PMC2703903

[20] Bolton EE Chen J Kim S. PubChem3D: a new resource for scientists. J Cheminf. 2011;3:32. 10.1186/1758-2946-3-32 PMC3269824 21933373

[21] Daina A Michielin O Zoete V. SwissTargetPrediction: updated data and new features for efficient prediction of protein targets of small molecules. Nucleic Acids Res. 2019;47:W357–64. 31106366 10.1093/nar/gkz382 PMC6602486

[22] Wishart DS Knox C Guo AC. DrugBank: a knowledgebase for drugs, drug actions and drug targets. Nucleic Acids Res. 2008;36:D901–906. 18048412 10.1093/nar/gkm958 PMC2238889

[23] Clough E Barrett T. The gene expression omnibus database. Methods Mol Biol. 2016;1418:93–110. 27008011 10.1007/978-1-4939-3578-9_5 PMC4944384

[24] Barrett T Wilhite SE Ledoux P. NCBI GEO: archive for functional genomics data sets--update. Nucleic Acids Res. 2013;41:D991–995. 23193258 10.1093/nar/gks1193 PMC3531084

[25] Shannon P Markiel A Ozier O. Cytoscape: a software environment for integrated models of biomolecular interaction networks. Genome Res. 2003;13:2498–504. 14597658 10.1101/gr.1239303 PMC403769

[26] Zhou Y Zhou B Pache L. Metascape provides a biologist-oriented resource for the analysis of systems-level datasets. Nat Commun. 2019;10:1523. 30944313 10.1038/s41467-019-09234-6 PMC6447622

[27] Szklarczyk D Gable AL Lyon D. STRING v11: protein-protein association networks with increased coverage, supporting functional discovery in genome-wide experimental datasets. Nucleic Acids Res. 2019;47:D607–13. 30476243 10.1093/nar/gky1131 PMC6323986

[28] Chin CH Chen SH Wu HH Ho CW Ko MT Lin CY. cytoHubba: identifying hub objects and sub-networks from complex interactome. BMC Syst Biol. 2014;8(Suppl 4):S11. 25521941 10.1186/1752-0509-8-S4-S11 PMC4290687

[29] Xu H Cao WZ Bai YY. Effects of sodium-glucose cotransporter 2 inhibitors on cardiovascular outcomes in elderly patients with comorbid coronary heart disease and diabetes mellitus. J Geriatr Cardiol. 2021;18:440–8. 34220973 10.11909/j.issn.1671-5411.2021.06.001 PMC8220386

[30] Björkegren JL Hägg S Talukdar HA. Plasma cholesterol-induced lesion networks activated before regression of early, mature, and advanced atherosclerosis. PLoS Genet. 2014;10:e1004201. 24586211 10.1371/journal.pgen.1004201 PMC3937269

[31] Price DT Loscalzo J. Cellular adhesion molecules and atherogenesis. Am J Med. 1999;107:85–97. 10403357 10.1016/s0002-9343(99)00153-9

[32] Wu W Wages PA Devlin RB Diaz-Sanchez D Peden DB Samet JM. SRC-mediated EGF receptor activation regulates ozone-induced interleukin 8 expression in human bronchial epithelial cells. Environ Health Perspect. 2015;123:231–6. 25303742 10.1289/ehp.1307379 PMC4348738

[33] Sluiter TJ van Buul JD Huveneers S Quax PHA de Vries MR. Endothelial barrier function and leukocyte transmigration in atherosclerosis. Biomedicines. 2021;9:328. 33804952 10.3390/biomedicines9040328 PMC8063931

[34] Maiuri MC Zalckvar E Kimchi A Kroemer G. Self-eating and self-killing: crosstalk between autophagy and apoptosis. Nat Rev Mol Cell Biol. 2007;8:741–52. 17717517 10.1038/nrm2239

[35] Kaplan O Demircan G. Relationship of autophagy and apoptosis with total occlusion of coronary arteries. Med Sci Monit. 2018;24:6984–8. 30273932 10.12659/MSM.910763 PMC6180916

[36] Abbate A Biondi-Zoccai GG Bussani R. Increased myocardial apoptosis in patients with unfavorable left ventricular remodeling and early symptomatic post-infarction heart failure. J Am Coll Cardiol. 2003;41:753–60. 12628718 10.1016/s0735-1097(02)02959-5

[37] Abbate A Melfi R Patti G. Apoptosis in recent myocardial infarction. Clin Ter. 2000;151:247–51. 11107673

[38] Nilsson L Szymanowski A Swahn E Jonasson L. Soluble TNF receptors are associated with infarct size and ventricular dysfunction in ST-elevation myocardial infarction. PLoS One. 2013;8:e55477. 23405158 10.1371/journal.pone.0055477 PMC3566185

[39] Crampon K Giorkallos A Deldossi M Baud S Steffenel LA. Machine-learning methods for ligand-protein molecular docking. Drug Discov Today. 2022;27:151–64. 34560276 10.1016/j.drudis.2021.09.007

[40] Moppel I Elliott B Chen S. Intermolecular hydrogen bonding behavior of amino acid radical cations. Organ Biomol Chem. 2024;22:3966–78. 10.1039/d4ob00301b 38690804

[41] Liao HW Hsu JM Xia W. PRMT1-mediated methylation of the EGF receptor regulates signaling and cetuximab response. J Clin Invest. 2015;125:4529–43. 26571401 10.1172/JCI82826 PMC4665782

[42] Aceves SS. Remodeling and fibrosis in chronic eosinophil inflammation. Dig Dis. 2014;32:15–21. 24603375 10.1159/000357004 PMC4037288

[43] Zhang MZ Wang Y Paueksakon P Harris RC. Epidermal growth factor receptor inhibition slows progression of diabetic nephropathy in association with a decrease in endoplasmic reticulum stress and an increase in autophagy. Diabetes. 2014;63:2063–72. 24705402 10.2337/db13-1279 PMC4030104

[44] Zhang Y Wang D Zhao Z. Nephronectin promotes cardiac repair post myocardial infarction via activating EGFR/JAK2/STAT3 pathway. Int J Med Sci. 2022;19:878–92. 35693734 10.7150/ijms.71780 PMC9149649

