# Supplementary material for: Bioinformatics study of the pharmacological mechanism of sodium-glucose co-transporter 2 inhibitors in type 2 diabetes mellitus and coronary heart disease based on network pharmacology
Source: Medicine (Baltimore). 2026 Jan 30;105(5):e47306. doi: 10.1097/MD.0000000000047306 (PMC12863777; doi:10.1097/MD.0000000000047306)

**Supplementary Material Figure S1: Histogram of expression fold changes of EGFR, UTGB2, and LCK in T2DM and CHD datasets.**

The horizontal axis represents genes, and the vertical axis represents the Log_2_FC value of genes.


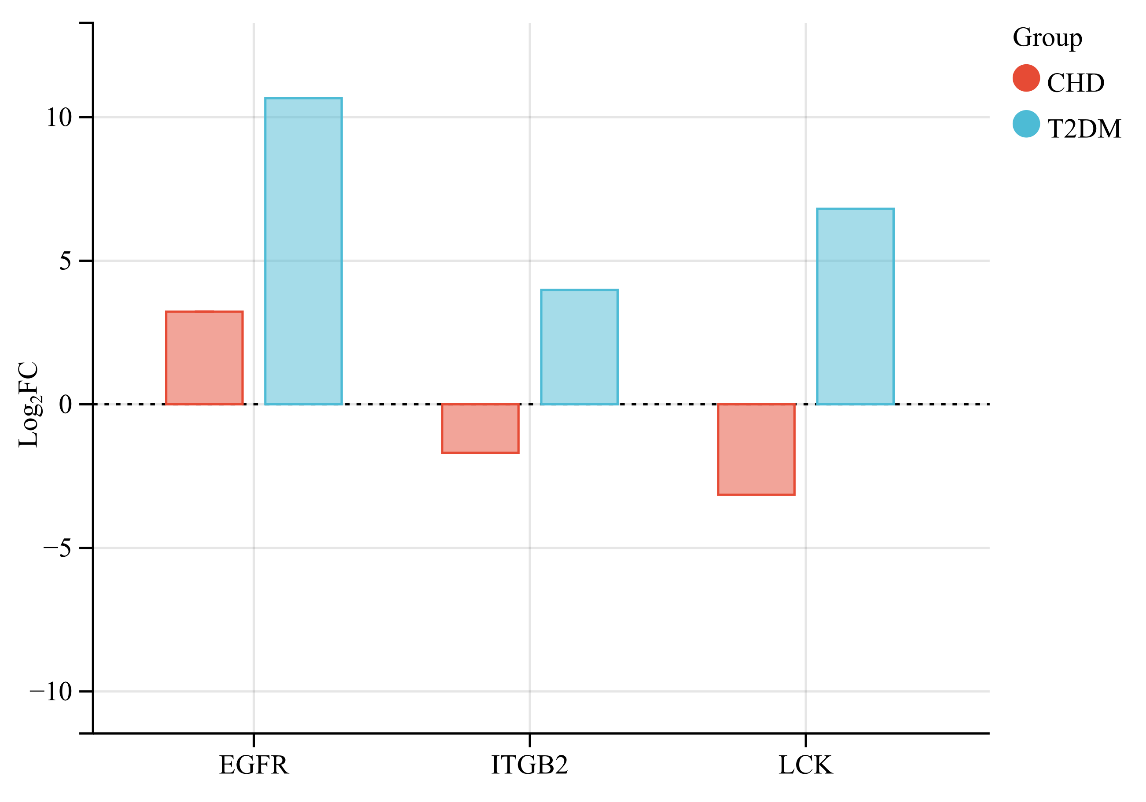

Supplement: Supplementary file 1 [file medi-105-e47306-s001.docx]
